# Snack food and beverage consumption and young child nutrition in low‐ and middle‐income countries: A systematic review

**DOI:** 10.1111/mcn.12729

**Published:** 2019-06-21

**Authors:** Alissa M. Pries, Suzanne Filteau, Elaine L. Ferguson

**Affiliations:** ^1^ Helen Keller International New York New York; ^2^ Department of Population Health, Faculty of Epidemiology and Population Health London School of Hygiene & Tropical Medicine London UK

**Keywords:** complementary feeding, double burden, low‐ and middle‐income countries, nutrition, snacks, sugar‐sweetened beverages

## Abstract

Although snacks can provide important nutrients for young children during the complementary feeding period, the increasing availability of snack foods and sugar‐sweetened beverages (SSB), often energy‐dense and nutrient‐poor, in low‐ and middle‐income countries (LMIC) is a concern. Such foods may displace consumption of nutritious foods in contexts where diets are often nutritionally inadequate and the burden of childhood malnutrition is high. This systematic review summarizes literature on the contribution of snack food/SSB consumption to total energy intakes (TEI) of children below 23 months of age in LMIC and associations between this consumption and nutritional outcomes. It also identifies areas where further research is needed. A systematic search of Embase, Global Health, and MEDLINE for literature published in January 1990–July 2018 was conducted. This search yielded 8,299 studies, 13 of which met inclusion criteria: Nine studies assessed % TEI from snack foods/SSB, and four studies assessed associations between snack food/SSB consumption and nutritional outcomes. Average % TEI from snack foods/SSB ranged from 13% to 38%. Findings regarding associations with growth were inconclusive, and no studies assessed associations with nutrient intakes. Variation in measurement of consumption and definitions of snack foods and SSB limited study comparisons. Further research is needed to understand how consumption of energy‐dense, nutrient‐poor snack foods and SSB influences undernutrition and overnutrition among young children during the complementary feeding period in settings that are experiencing dietary transitions and the double burden of malnutrition.

**Key messages**

- Limited evidence suggests that snack foods and sugar‐sweetened beverages (SSB) are providing a substantial proportion of energy intakes among children below 2 years of age in Latin American and South‐east Asian LMIC.
- There is limited and inconclusive evidence on relationships between snack food/SSB consumption patterns and young children's overall dietary adequacy, micronutrient status, and growth. Given that diets in LMIC are often nutrient‐poor and young children's nutrient requirements are high, the high consumption of such foods may contribute to undernutrition, as well as increased risk of overnutrition, and more research is needed.
- Definitions of snack foods/SSB and measurement of their consumption among children are wide ranging. There is a need for standardized approaches in order to allow comparison between regions and over time, as well as a need to expand research on contribution of snack foods/SSB to young children's diets and nutritional outcomes in regions where malnutrition is highest.

## 1 INTRODUCTION

Ensuring a nutritious diet in the first 2 years of life, in terms of both quantity and quality, is vital for young children's nutrition and health (Shrimpton, Victora, De Onis, Lima, & Blo, 2001; World Health Organization, 1998). During this period of accelerated growth and development, a child's nutrient requirements are high. It is therefore recommended to introduce appropriate and nutrient‐rich complementary foods, including snacks, at 6 months of age while continuing to breastfeed. The types of snacks fed to young children however are important to ensure diet quality. Snack food products and sugar‐sweetened beverages (SSB) are often energy‐dense, nutrient‐poor, and high in salt or sugar (Lucan, Karpyn, & Sherman, 2010; Monteiro, Levy, Claro, de Castro, & Cannon, 2011; Sekiyama, Roosita, & Ohtsuka, 2012; Waseem et al., 2014), making them inappropriate for infant and young child feeding (Moodie et al., 2013).

The growing availability of unhealthy processed foods in many low‐ and middle‐income countries (LMIC) is a concerning trend (Monteiro, Moubarac, Cannon, Ng, & Popkin, 2013). Overconsumption of snack food products and SSB has been shown to contribute to overweight and obesity among children in the United States (Nicklas, Yang, Baranowski, Zakeri, & Berenson, 2003; Phillips et al., 2004; Scientific Advisory Committee on Nutrition, 2014; Welsh, 2005) and Latin America (Asfaw, 2011; Rauber, Campagnolo, Hoffman, & Vitolo, 2015; Tavares, Fonseca, Garcia Rosa, & Yokoo, 2012). Additionally, energy‐dense, nutrient‐poor foods early in life can displace consumption of other nutritious foods, including breast milk (Kimmons et al., 2005), potentially increasing a child's risk of inadequate nutrient intakes and contributing to childhood undernutrition. The correlation between consumption of snack foods/SSB and lower consumption of nutrient‐rich foods and/or reduced nutrient intakes has been shown in high‐income settings (Bhargava & Amialchuk, 2007; Bowman, 1999; Kant, 2003; Marriott, Olsho, Hadden, & Connor, 2010; Murakami & Livingstone, 2015; Rennie & Livingstone, 2007; Webb et al., 2006).

Prevalent consumption of snack foods and SSB among young children has been noted across Africa, Asia, and Latin America (Bentley et al., 2015; Faber & Benade, 2007; Pantoja‐Mendoza, Melendez, Guevara‐Cruz, & Serralde‐Zuniga, 2014; Pries et al., 2017; Woo et al., 2013). However, the role these foods play in the overall diets of infants and young children in LMIC, as well as their impact on their nutrition, remains unclear. The influence of such foods on diet quality and nutritional outcomes is hypothesized to be different in LMIC, as compared with high‐income settings, given the higher burden of undernutrition and limited accessibility of nutrient‐dense foods in diets. The purpose of this systematic review was therefore to synthesize available literature on the contribution of snack food and SSB to total energy intakes (TEI) among children 0–23 months of age in LMIC and associations between consumption of such foods/beverages and nutritional status of young children in these settings, as well as to identify future research needs within this topic area.

## 2 METHODS

### 2.1 Search terms and study selection

**Figure 1:**
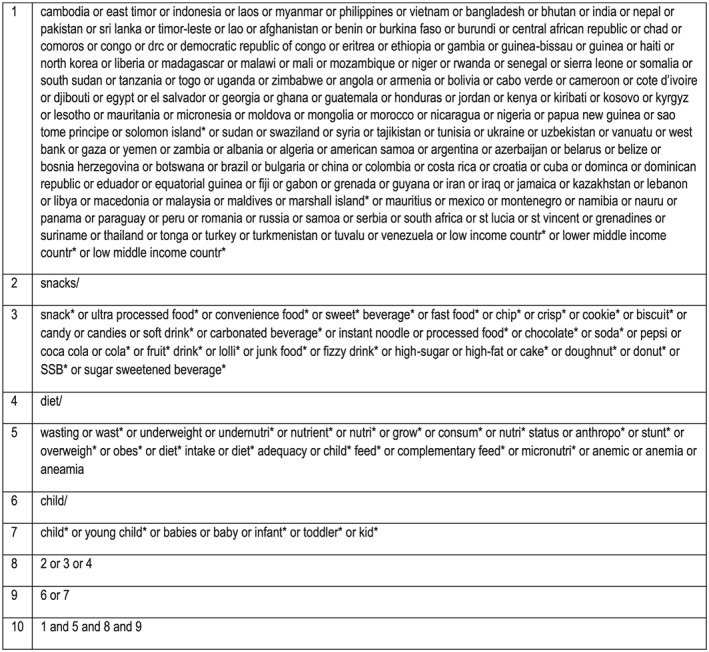
Search terms

A systematic search was conducted across three databases on July 25, 2018: Embase, Global Health, and MEDLINE, using the search terms presented in Figure 1. These terms were based on four broad categories: (a) low, lower‐middle, or upper‐middle‐income countries based on World Bank (2017) classifications, (b) energy‐dense, nutrient‐poor snack foods and beverages, (c) diet or nutrition, and (d) children. Because definitions for snack foods and SSB are wide ranging, the search strategy identified all studies related to children's diets, which were then screened to identify those that included specific consumption measurement of snack foods/SSB; this included measurement of types of foods (e.g., biscuits, candy, and soft drinks) or categories of foods (ultraprocessed foods, discretionary foods, snack foods, junk foods, noncore foods, etc.). Titles and abstracts were screened first for exclusion/inclusion, which was followed by full‐text screening. All three researchers reviewed search strategy and terms (AP, SF, and EF), and screening was conducted by one researcher (AP). Screened studies were included if they met the following criteria: (a) They were conducted in an LMIC, (b) the study population included children below two years of age, and (c) they assessed the contribution of snack foods and/or SSB to children's TEI (based on kilocalories) or the association between children's consumption of snacks foods and/or SSB and nutrient intakes/micronutrient status/anthropometric status. Studies were excluded based on the following criteria: (a) studies published prior to January 1, 1990 (based on the assumption that availability and use of snack foods/SSB in LMIC have changed in the last two to three decades), (b) literature not published in English, (c) results published as conference/meeting abstracts only, (d) studies that assessed contribution to TEI from added sugars only, or (e) studies with a wider age range than children 0–23 months of age that did not present specific data within this age range. References of included studies were also hand‐searched to identify relevant studies for inclusion; no additional studies were identified through this process.

### 2.2 Data extraction and synthesis

For studies presenting data on the proportion of TEI derived from snack foods and/or SSB, the following information was extracted: reference, study population, sample size, location, study design, dietary assessment methods, diet findings, and their definition of snack food/SSB. For studies that tested associations between snack food/SSB consumption and child nutritional status, the following additional information was extracted: the nutritional outcomes tested and results of the associations tested.

## 3 RESULTS

**Figure 2:**
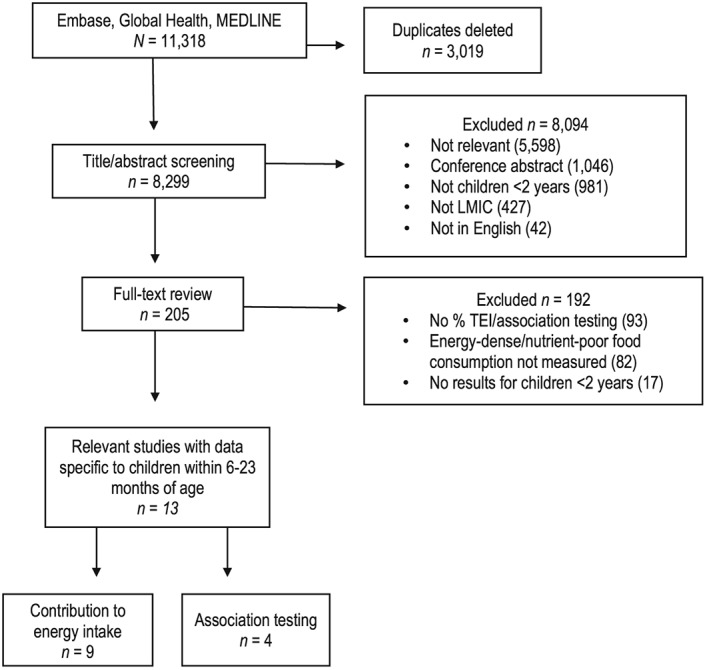
Flow diagram of study selection. LMIC: low‐ and middle‐income countries; TEI: total energy intake

After deletion of 3,019 duplicates, this search resulted in 8,299 studies. After title/abstract screening, 205 studies were identified as relevant for full‐text review. The majority of studies were excluded because the study population did not include children below two years of age. During full‐text review, 30 relevant studies were identified with age ranges that included children 0–23 months of age in their samples. Just under half of these studies however presented data on children specifically within the 0–23 months of age range. A total of 13 studies met the selection criteria: Nine studies detailed the proportion of energy intake derived from snack foods or SSB, and four examined associations between snack food/SSB consumption and nutritional status (Figure 2).

### 3.1 Contribution to energy intake

Nine studies identified in this review assessed the contribution of snack foods/SSB to dietary energy intakes among children within the complementary feeding period. Details of these studies are presented in Table 1. Five of these studies were from Latin America, three studies were from East/South‐east Asia, and one study was from Egypt.

**Table 1:** Summary of studies assessing contribution of snack food/SSB consumption to total energy intake

Reference	Age, sample size, location	Dietary assessment method	Food/beverage	Snack food/SSB definition	Snack food/SSB consumption
Anderson et al. (2008)	12–42 months (*N* = 210) Subanalysis: 12–23 months (*n* = 61) Cambodia (Phnom Penh, urban)	Quantitative 24HR (1 day)	Unclear	Snack foods (definition not provided)	• Snack food products were the predominant source of energy for partially breastfed (42% TEI) and nonbreastfed (36% TEI) children 12–23 months of age • 38.2% TEI from snacks/SSB among all 12‐ to 23‐month‐olds
Denney et al. (2017)	0–48 months (*N* = 2057) Subanalysis: 6–23 months (*n* = 767) Mexico (national)	Quantitative 24HR (1 day)	Food and beverage	Sweets: Cookies, cakes, pies/pastries, sweetened breads, candy, Mexican desserts, ice cream, sugars, syrups, jelly, fruit drinks, soft drinks, sweetened tea/coffee, artificially sweetened beverages, Yakult, sweet traditional beverages; Salty snacks: Grain snacks and those made from starchy vegetables	• Among 6‐ to 11‐month‐olds, 4.3% of TEI from cookies, 1.7% from sweet traditional beverages, 1.0% from sweetened breads, and 1.0% from salty snacks; among 12‐ to 23‐month‐olds, 4.9% of TEI from sweetened breads, 4.7% from sweet traditional beverages, 3.9% from cookies, 2.6% from sweetened tea/coffee, 2.2% from salty snacks, and 1.3% from fruit‐flavoured drinks • 16.1% TEI from snacks/SSB among 6‐ to 23‐month‐olds
Jeharsae et al. (2011)	1–5 years (*N* = 478) Subanalysis: 12–23 months (n not provided) Thailand (conflict area)	Quantitative 24HR (1 day)	Unclear	Definition not provided	• Snacks accounted for 19.3% of TEI among children 12–23 months old
Karnopp et al. (2017)	1–72 months (*N* = 770) Subanalysis: <24 months, nonexclusive breastfeeding (*n* = 214) Brazil (Pelotas, urban)	Quantitative 24HR (1 day)	Food and beverage	Ultraprocessed foods: Bread, cakes and baked products, cookies, ice cream, chocolates, candies and sweets in general; cereal bars, breakfast cereals with added sugar, sweetened and flavoured yogurt and dairy beverages; energy drinks; frozen and ready‐to‐heat foods (pasta, pizza, and burgers), nuggets, frankfurters and sausages, and pre‐prepared dishes and sauces; hydrogenated vegetable fat (margarine and halvarine), chips; sauces; sweet and savoury snacks; soft drinks and processed juices; canned meat and dehydrated soups; ready‐made noodles; infant formula, complementary formula, and processed baby food; and artificial sweeteners	• 19.7% of TEI from ultraprocessed foods among children <24 months: 12.9% of TEI from “others foods” (industrialized juice, processed baby food, supplements and powdered infant formula), 2.6% from cookies, 1.9% from bread, and 1.8% from candies/sweets • 19.7% TEI from snacks/SSB among nonexclusively breastfed <24‐month‐olds
Kavle et al. (2015)	6–23 months (*N* = 120) Egypt (Qaliobia, periurban; Sohag, rural)	Quantitative 24HR (1 day)	Food and beverage	Junk foods: High energy, low in nutrient content and/or high in fat and/or contain added sugar (sugary biscuits, cream‐filled sponge cakes, candy, fizzy drinks) or have high salt content (crisps/chips)	• 20.9% of TEI came from junk foods among 6‐ to 8‐month‐olds, 18.8% among 9‐ to 11‐month‐olds, and 9.0% among 12‐ to 23‐month‐olds • 14.3% TEI from snacks/SSB among 6‐ to 23‐month‐olds
Lander et al. (2010)	6–23 months (*N* = 128) Mongolia (Ulaanbaatar and four provincial capitals, urban)	Quantitative 24HR (1 day)	Unclear	Snacks and sugars: “Mainly doughnuts and biscuits” (definition not provided)	• Among 6‐ to 8‐month‐olds, 27% of TEI came from snacks, 35% of TEI for 9‐ to 11‐month‐olds, and 40% of TEI for 12‐ to 23‐month‐olds
Roche et al. (2011)	0–23 months (*N* = 32) Peru (Amazonas district, rural)	Quantitative 24HR (2 days, nonconsecutive)	Unclear	Market foods; packaged and commercially sold (definition not provided)	• 13.1% TEI from market foods
Rodríguez‐Ramírez et al. (2016)	0–23 months (*N* = 926) Subanalysis 6–23 months (*n* = 749) Mexico (national)	Quantitative 24HR (1 day)	Food and beverage	Dairy SSB: Milk shake, atole with milk, milk with sugar/honey Non‐dairy SSB: Beverages prepared with water and fruit or its juice (natural or industrialized) and sugar/honey, sodas, carbonated beverages, soft drinks with calorie sweeteners, fruit juices (natural and industrialized), coffee/tea/infusion/water with sugar/honey, atole with water Sweet cereals/bread/cookies: Oats, tapioca, milk pudding, granola bars, fresh bread and bakery, cakes, cookies, pastries, desserts Snacks and desserts: Chips, fried snacks made of wheat flour, candies, gummies, lollies, ice cream/popsicles, jam, marmalade	• Among 12‐ to 23‐month‐olds, approximately 10% of TEI from sweetened cereal foods, 3% from snacks and desserts, 5% from non‐dairy SSB, and 5% from dairy SSB (exact proportions not clear in figures presented) • Approximately 20% TEI from snacks/SSB among 6‐ to 23‐month‐olds (exact proportions not clear in figures presented)
Valmórbida and Vitolo (2014)	12–16 months *N* = 388 Brazil (Porto Alegre, urban)	Quantitative 24HR (two nonconsecutive days)	Food and beverage	Nonrecommended foods: Candies, lollipops, chocolates, cookies, jello, petit suisse cheese, chocolate milk, sausages, snacks, soft drinks, artificial juices, and foods with added sugar	• 13.6% of TEI from nonrecommended foods

*Note*. 24HR: 24‐hr recall; TEI: total energy intake; SSB: sugar‐sweetened beverage.

The reported % TEI ranged from 13.1% among 0‐ to 23‐month‐olds in the Amazonas district of Peru (Roche, Creed‐Kanashiro, Tuesta, & Kuhlein, 2011) to 38.2% among 12‐ to 23‐month‐olds in Phnom Penh, Cambodia (Anderson, Cornwall, Jack, & Gibson, 2008), with a median of 19.3% TEI across all nine studies. Five studies assessed dietary energy contribution from both snack foods and SSB (Denney, Afeiche, Eldridge, & Villalpando‐Carrión, 2017; Karnopp et al., 2017; Kavle et al., 2015; Rodríguez‐Ramírez, Muñoz‐Espinosa, Rivera, González‐Castell, & González de Cosío, 2016; Valmórbida & Vitolo, 2014). Four studies did not specifically indicate if they assessed contributions from SSB in addition to snack foods (Anderson et al., 2008; Jeharsae, Sangthong, & Chongsuvivatwong, 2011; Lander et al., 2010; Roche et al., 2011).

Four studies presented differences in % TEI from snacks/SSB across age groups, with most showing an increase in % TEI from such foods among older children. Across the entire complementary feeding period, Lander et al. (2010) and Denney et al. (2017) found that % TEI from snack foods and SSB increased with age (27.0% among 6‐ to 8‐month‐olds vs. 35.0% among 9‐ to 11‐month‐olds vs. 40.0% among 12‐ to 23‐month‐olds; and 8.0% among 6‐ to 11‐month‐olds vs. 19.6% among 12‐ to 23‐month‐olds, respectively). However, a decrease in % TEI from processed snack foods/SBB with age was shown by Kavle et al. (2015) in rural and periurban Egypt.

### 3.2 Relationships between consumption and nutritional outcomes

Four studies assessed relationships between consumption of snack foods/SSB and nutritional outcomes among children during the complementary feeding period in LMIC (Table 2). Three studies looked at associations with anthropometry, one study looked at the association with anaemia, and no studies reported association testing between snack food/SSB consumption and dietary nutrient intakes. These four studies were conducted in countries that spanned three separate regions, with three conducted in urban/periurban locations and one in a rural location (Faber, 2007). Three studies included consumption of both snack foods and SSB in their analyses, whereas one study included consumption of snack foods only (not SSB; Vakili, Kiani, Saeidi, Hoseini, & Anbarani, 2015).

**Table 2:** Summary of studies testing associations between snack food/SSB consumption and nutritional outcomes

Reference	Age, sample size, location, study design	Dietary intake assessment method	Food/beverage focus	Comparison group	Statistical method	Snack food/SSB definition	Nutritional outcome	Direction of association (*P* value)
Budree et al. (2017)	6–12 months (*N* = 1,071) South Africa (Paarl, periurban) Cohort	Questionnaire: Frequency of consumption in previous day, week, and month	Food and beverage	Consumption of inappropriate foods daily versus no consumption of inappropriate foods daily	Linear regression	Inappropriate foods: Juices, soft drinks, sugary foods, fried foods	BMIZ HAZ WAZ MUACZ (at 12 months)	NS NS NS NS
Faber (2007)	6–12 months (*N* = 479) South Africa (KwaZulu‐Natal, rural) Cross‐sectional	Questionnaire: Unquantified frequency of consumption in the previous week	Food and beverage	Consumption of food types at least 4 days per week	*χ* ^2^ test	Miscellaneous foods: Sugar, biscuits, sweets, savoury snacks, and carbonated beverages	Anaemia (haemoglobin concentration < 100 g L^−1^)	NS
Jimenez‐Cruz et al. (2010)	5–24 months (*N* = 810) Mexico (Tijuana, Tuxtla, and Reynosa, urban) Cross‐sectional	Questionnaire: Frequency of consumption in the previous week	Food and beverage	Consumption of high‐fat content snacks and/or sweetened drinks at least once in the previous week versus no consumption	Logistic regression	High‐fat snacks (HFS;i.e., potato and corn ships) and carbonated/noncarbonated sweetened drinks (CSD)	Overweight/obese (BMIZ > 2)	+(<0.001)
Vakili et al. (2015)	6–24 months (*N* = 300) Iran (Mashhad, urban) Cross‐sectional	Questionnaire: Use of junk food for child feeding (definitions of regular use and sometimes use not provided)	Food	Use of junk foods for child feeding versus non‐use of junk foods	*χ* ^2^ test	Junk food: Definition not provided	Growth delay (definition not provided)	+(<0.001)

*Note*. NS: not significant; BMIZ: body mass index *z*‐score; HAZ: height‐for‐age *z*‐score; WAZ: weight‐for‐age *z*‐score; MUAC: mid upper arm circumference.

For associations between snack food/SSB consumption and anthropometric outcomes, one study assessed differences in mean *z*‐scores (Budree et al., 2017), one study assessed associations with overweight/obesity, and one study assessed associations with growth delay (Vakili et al., 2015). Budree et al. (2017) found no relationship between snack food/SSB consumption and body mass index *z*‐score (BMIZ), height‐for‐age *z*‐score (HAZ), or weight‐for‐age *z*‐score (WAZ), comparing mean *z*‐scores among 12‐month‐olds who had consumed snack foods/SSB daily with those who consumed these foods less frequently (BMIZ *β* = −0.01, 95% CI [−0.4, 0.4]; HAZ *β* = 0.2, 95% CI [−0.3, 0.6]; WAZ *β* = 0.1, 95% CI [−0.5, 0.5]). Jimenez‐Cruz, Bacardi‐Gascon, Pichardo‐Osuna, Mandujano‐Trujillo, and Castillo‐Ruiz (2010) noted 1.87 higher odds of overweight/obesity (BMIZ > 2) among 5‐ to 24‐month‐olds who consumed high‐fat snack foods and SSB at least once a week; this relationship was also noted for consumption of SSB only (OR: 1.62, 95% CI [1.10, 2.36]) and consumption of high‐fat foods only (OR: 1.91, 95% CI [1.31, 2.78]). The study in Iran by Vakili et al. (2015) noted a positive association between regular feeding of junk food among 6‐ to 24‐month‐olds and growth delays. The definition and measurement of growth delay however were not presented in the paper. One study (Faber, 2007) assessed the association between consumption of various types of snack foods/SSB—biscuits, sweets, savoury snacks, or soft drinks—and anaemia among 6–12 months of age in rural South Africa, with no statistical differences in proportions of anaemic versus nonanaemic children noted.

## 4 DISCUSSION

This review indicates that the % TEI contributed by snack foods and SSB among children in the 0‐ to 23‐month age range in LMIC ranged from 13% in rural Peru to 38% in urban Cambodia. Evidence regarding the influence of snack food and SSB consumption on children's dietary adequacy and nutritional status in these contexts is however limited. Results from the three studies that explored associations between snack food/SSB consumption and child growth outcomes show mixed findings: one found no significant relationship with *z*‐scores (Budree et al., 2017), one found a positive relationship with child overweight/obesity (Jimenez‐Cruz et al., 2010), and one identified a positive relationship with child growth delays (Vakili et al., 2015). No studies were identified that assessed associations between snack food/SSB consumption and dietary nutrient intakes, and only one assessed child micronutrient status, specifically, anaemia status.

Although this review indicates that snack foods/SSB are potentially providing a substantial proportion of dietary energy among young children in LMIC, the low number of studies and their limited geographical distribution limit the ability to understand whether the % TEI from snack foods/SSB differs between urban versus rural populations and across regions, particularly those beyond Latin America and East/South‐east Asia. Of the nine studies that explored % TEI from snack foods/SSB, four were conducted in urban contexts (Anderson et al., 2008; Karnopp et al., 2017; Lander et al., 2010; Valmórbida & Vitolo, 2014), and one was conducted in a conflict area (Jeharsae et al., 2011). Two studies used national datasets (Denney et al., 2017; Rodríguez‐Ramírez et al., 2016); however, neither disaggregated data among 0‐ to 23‐month‐olds by rural/urban area of residence. Kavle et al. (2015) and Roche et al. (2011) presented findings for rural samples of children; however, the small sample sizes (*n* = 60 and *n* = 32, respectively) likely limit the precision of the % TEI results for these subpopulations and the ability to generalize results. Of all nine studies assessing contributions of energy intake from snack foods and SSB, the majority were from Latin America (specifically Peru, Mexico, or Brazil; *n* = 5) and East/South‐east Asia (Cambodia, Indonesia, or Mongolia; *n* = 3). There were no studies from South Asia, and only one study was conducted in northern Africa (none were conducted in southern or eastern Africa), revealing a dearth of information in contexts where the global burden of undernutrition is highest. Additionally, only three studies assessing % TEI considered variation across age groups, with two noting an increase in % TEI from snack foods/SSB and one noting a decrease, indicating that the function of these foods spanning the complementary feeding period could differ across regions of the world. There is a need for more research to explore the contribution of snack foods and SSB to energy intakes in the diets of infants and young children in LMIC, extending geographic regions, assessing urban versus rural areas, and exploring age trends.

The median % TEI from processed snack foods/SSB for children below 23 months across studies in this review was 19%, with a range of 13–38%. Percent TEI from snack foods/SSB among adolescents in LMIC, as well as among children in high‐income settings, has also been noted within this range. Among Malaysian adolescents, 24% of TEI came from snack foods (Boon, Sedek, & Kasim, 2012), and among Filipino 15‐year‐olds, 21% TEI came from snack foods (Adair & Popkin, 2005). In high‐income settings, 31% and 27% TEIs came from snack foods/SSB among 2‐ to 6‐year‐olds in Russia and the United States, respectively (Adair & Popkin, 2005), and 31% of TEI among American children and adolescents 8–18 years of age came from low‐nutrient density foods, such as processed snack foods and SSB (Kant, 2003). Although high consumption of snack foods and SSB has often been thought to be a problem specific to school‐age children and children in higher socio‐economic settings, the findings from this systematic review suggest that these foods are now making up a significant portion of total dietary intake among infants and young children in low‐income settings in some regions of the world.

This systematic review also identified a need for further research to examine the relationship between snack food/SSB and dietary nutrient intake adequacy during the crucial complementary feeding period in LMIC. Although no studies in this review explored the relationship between snack food/SSB consumption and micronutrient intakes, based on the average % TEI noted, it is plausible that such consumption patterns are contributing to reduced dietary nutrient intakes among young children in LMIC settings. There is increasing evidence that high intakes of energy‐dense, nutrient‐poor snack foods/SSB contribute to micronutrient dilution and reduced nutrient intakes among adolescents and adults (Louzada et al., 2015). In a systematic review of evidence evaluating the nutritional significance of added sugar consumption, Gibson (2007) concluded that very high intakes of added sugars (over 20% of energy intake)—particularly when consumed in the form of soft drinks, sugar, and sweets—are correlated with lower intakes of some micronutrients among school‐age children in high‐income settings. Among U.S. children 8–18 years of age, Kant (2003) found that mean intakes of vitamins A and B6 and folate, as well as calcium, magnesium, iron, and zinc, all declined with increased consumption of low‐nutrient‐dense foods (candy, baked and dairy desserts, salty snacks, and SSB), with these foods contributing 30% TEI on average in the study sample. Among Australian 16‐ to 24‐month‐olds, Webb et al. (2006) noted reduced intakes of many nutrients, including calcium, zinc, and vitamin A, among the highest consumers of snack foods/SSB, with these foods contributing 27% TEI on average. Among South African 1‐ to 3‐year‐olds, those in the highest quartile of added sugar consumption (based on % TEI) had lower intakes of calcium, iron, and zinc, as compared with toddlers with lower % TEI from added sugar (Maunder, Nel, Steyn, Kruger, & Labadarios, 2015). Five studies in this review (Anderson et al., 2008; Jeharsae et al., 2011; Karnopp et al., 2017; Lander et al., 2010; Rodríguez‐Ramírez et al., 2016) noted % TEI from snack foods/SSB of approximately 20% or higher among children below 23 months of age, suggesting levels of consumption that could contribute to micronutrient dilution. It is also critical to note that this review did not identify any studies that explored the relationship between snack food/SSB consumption and reduced micronutrient intakes, dietary adequacy, or micronutrient status beyond anaemia. There is a clear need for further research on this pathway, given that young children have high nutrient requirements and the nutrient density of complementary foods in LMIC is often low (Faber, Laubscher, & Berti, 2016; Kimmons et al., 2005).

Findings regarding the association between snack food/SSB consumption and growth outcomes were limited and mixed among studies in this review. The study by Budree et al. (2017) did not find any significant relationships with BMIZ, HAZ, or WAZ. However, the researchers did not assess % TEI from snack foods/SSB and instead based analysis on nonquantitative measurements of snack foods/SSB consumption, which may not be able to precisely estimate consumption levels associated with micronutrient dilution and/or excessive energy intakes and that may be necessary to establish a relationship between consumption and growth outcomes. Additionally, the population in this study was 6–12 months of age; as seen in the studies detailing % TEI from snacks, the contribution of such foods to total dietary intake tends to be higher among older children. It may be that the quantity of snack food/SSB consumption at 6–12 months was not substantial enough to result in an impact on growth outcomes. Jimenez‐Cruz et al. (2010) found increased odds of overweight/obesity among Mexican 5‐ to 24‐month‐olds, whereas Vakili et al. (2015) found a higher prevalence of growth delay among Iranian children 6–24 months of age who ate snacks. The influence of such foods could in theory contribute to either overnutrition, through excessive energy intakes, or undernutrition, through displaced consumption of nutrient‐dense foods, and so the findings from these two studies are not inherently contradictory. However, with only two studies, both of which used varying nonquantitative measurements of snack food/SSB consumption, it is difficult to draw conclusions based on these limited results. The biological/nutritional significance of diet displacement/micronutrient dilution from snack foods and SSB is not yet clear (Rennie & Livingstone, 2007), as it has not yet been established if such displacement translates into micronutrient deficiencies or growth faltering (Gibson, 2007).

The inconclusive evidence among studies in this review and evidence from high‐income settings indicate that more research on the potential impact of snack foods/SSB on growth among young children in LMIC is needed. Numerous studies among school‐age children/adolescents in LMIC, particularly South and South‐east Asia, have identified a relationship between snack food/SSB consumption and overweight/obesity (Elangovan, Mungara, & Joseph, 2012; Gonzalez‐Suarez et al., 2013; Goyal et al., 2010; Ishaque, Ahmad, Zehra, & Amin, 2012; Jain, Pant, Chopra, & Tiwari, 2010; Kumar & Faisal, 2015; Mistry & Puthussery, 2015; Tang et al., 2010) and waist circumference (Kuriyan et al., 2012). A positive association between SSB consumption and overweight/obesity among preschoolers in the United States has also been noted (Welsh, 2005), whereas another U.S. study found an association between SSB consumption and growth faltering among preschoolers (Dennison, Rockwell, & Baker, 1997). The influence of such consumption patterns on growth outcomes would likely be different in LMIC contexts, where constrained diets are often nutrient‐poor, and among young children, whose nutrient requirements are high. Given these differing circumstances, micronutrient dilution from high consumption of energy‐dense/nutrient‐poor foods could plausibly contribute to micronutrient deficiencies and poor growth outcomes and requires further research.

Limitations in study design, particularly related to sampling and measurement, challenge the ability to draw conclusions across papers identified in this review. Among the nine studies assessing % TEI from snack foods/SSB, only two provided nationally representative estimates, both from Mexico (Denney et al., 2017; Rodríguez‐Ramírez et al., 2016). Three of the remaining studies assessed nonrandom samples, including convenience samples of stunted children and mothers attending health services (Anderson et al., 2008; Roche et al., 2011; Valmórbida & Vitolo, 2014), limiting conclusions from these papers to wider populations. The four studies testing associations with nutritional outcomes also did not utilize representative samples—three studies systematically sampled mothers attending health centres for child health/vaccination services (Budree et al., 2017; Jimenez‐Cruz et al., 2010; Vakili et al., 2015), and one included all children within catchment areas of selected health facilities (Faber, 2007). Additionally, limitations in measurement of exposure and outcomes are noted among the four studies testing associations between consumption and nutritional outcomes. First, none of these studies utilized a comparable measurement of consumption of snack foods/SSB; the four separate measurements of consumption included any consumption in the last week, consumption on at least 4 days in the previous week, daily consumption based on weekly recall, and “use” or “non‐use” of foods for child feeding. In addition to restricting comparability across the four studies, such measurements crudely capture consumption of snack foods and SSB and would not accurately estimate the magnitude of consumption that would theoretically influence nutritional outcomes. A comparable measure of consumption, specifically one that quantifies the intakes of snack foods/SSB such as % TEI, would aid investigations into the relationship between consumption and diet/nutritional outcomes. Finally, although two studies used standardized measurements and definitions for anthropometrics, the lack of definition of “growth delay” in the paper by Vakili et al. (2015) prohibits understanding of the study findings and comparison with other papers.

The wide range of definitions used for snack foods and SSB, in both studies testing associations and studies describing % TEI, is clear from this review. Varying definitions included: the NOVA classification (with further variation between studies on inclusion of processed and/or ultraprocessed foods; Moubarac, Parra, Cannon, & Monteiro, 2014), specific food types such as “desserts” and “candy,” and food categorization such as “sugary foods.” Additionally, three studies did not provide a specific definition of snack foods, or indicate if both foods and SSB were included in their definition. As diets continue to evolve in LMIC, there is a need for a standardized definition to allow for comparisons between geographic areas, rural and urban populations, and across time. Such a definition could include food/beverage types that are typically common across geographies (i.e., candies, biscuits, and soft drinks) but also context‐specific foods (e.g., instant noodles and *aguas frescas*) and could also differentiate snack foods versus SSB given differential trends in use for young child feeding. The underlying hypothesis for influence of these foods on diet/nutritional status is centred upon these foods being both energy‐dense and nutrient‐poor; therefore, it is also recommended that the nutrient profile of these foods be assessed when possible.

This review indicates that snack foods and SSB contribute a substantial proportion of dietary energy intakes among young children in LMIC. However, there is a need to standardize definitions of such foods and coordinate measurement in order to better understand the influence of these consumption patterns on nutritional outcomes. Though diet displacement and micronutrient dilution from processed foods and added sugars have been noted among children in high‐income settings, the impact of such dilution in contexts struggling with undernutrition may be significantly higher and may be contributing to childhood undernutrition. Additional studies exploring the relationship between % TEI from processed foods and SBB and nutritional outcomes—including nutrient intakes, micronutrient status, and growth—among representative samples of young children in LMIC, particularly in Asia and Africa, are needed to better understand this issue. As economies develop and food systems change, there is a timely need for further investigation into the role of these foods in child nutritional outcomes in order to protect and promote nutritious and appropriate young child feeding.

## CONFLICTS OF INTEREST

The authors declare that they have no conflicts of interest.

## CONTRIBUTIONS

AP conducted the literature review and developed the manuscript. EF and SF reviewed the search strategy and contributed to the development of the manuscript.
